# Determinants of post-penitentiary social reintegration: analysis of the needs and perceptions of persons released from detention in Romania

**DOI:** 10.3389/fpsyg.2025.1484889

**Published:** 2025-03-27

**Authors:** Denisa Ramona Chasciar, Vasile Chasciar, Claudiu Coman, Ovidiu Florin Toderici, Liviu Toader, Attila Kovacs, Maria Cristina Bularca

**Affiliations:** ^1^Faculty of Educational Sciences, Psychology, and Social Work, Babeș-Bolyai University of Cluj-Napoca, Cluj-Napoca, Romania; ^2^Faculty of Social Sciences, University of Craiova, Craiova, Romania; ^3^Faculty of Sociology and Communication, Transilvania University of Brașov, Brașov, Romania; ^4^Faculty of Educational Sciences, Psychology, and Social Sciences, Aurel Vlaicu University of Arad, Arad, Romania

**Keywords:** social reintegration, post-penitentiary, discrimination, community support, recidivism

## Abstract

**Introduction:**

The social reintegration of people released from prison represents a significant challenge in Romania, being influenced by factors such as discrimination, social marginalization, and economic difficulties.

**Methods:**

This study analyzes the needs and perceptions of 1,039 people released from detention, using data collected through a questionnaire and an interview guide.

**Results:**

The results highlight the main difficulties encountered, including lack of community support (30.3%), difficulties in returning to family and community (17.8%), lack of housing (8.9%), and lack of financial resources (27.5%). The study also highlights the importance of professional qualification and access to the labor market, with 13.6% of respondents reporting a lack of adequate qualification and 22.8% experiencing difficulties due to a criminal record. The comparative analysis of these difficulties shows the need for multidimensional interventions and support from public and private institutions.

**Discussion:**

The conclusions suggest the development of comprehensive public policies that address the complex needs of these individuals, including financial and housing support, vocational training programs, and anti-discrimination measures, to facilitate effective reintegration and reduce the rate of recidivism.

## 1 Introduction

The social reintegration of people released from prison represents a major challenge for contemporary Romanian society. The reintegration process is often influenced by a number of complex factors, including discrimination, social marginalization, economic hardship, and lack of adequate support from the community and public institutions (Petersilia, 2003; Western et al., 2015). These problems not only affect the chances of successful reintegration, but also contribute to an increase in the recidivism rate (Lattimore et al., 2016; Visher and Travis, 2011).

The social marginalization of people released from detention is a well-documented phenomenon. Their stigmatization significantly reduces the chances of employment and access to essential services, increasing the risk of recidivism (Pager, 2003; Solomon et al., 2008). Lack of access to stable housing is another critical factor that negatively affects social reintegration (Herbert et al., 2015).

The economic hardship faced by these individuals is often exacerbated by a lack of adequate professional qualifications and criminal record biases (Bushway et al., 2007; Holzer et al., 2006). Also, having a criminal record considerably reduces the likelihood of getting a job, leading to economic and social instability (Pager et al., 2009).

In Romania, the socio-economic context and the legislative framework significantly influence the reintegration process of former detainees. Social support policies and vocational training programs are often insufficient to meet the complex needs of this vulnerable category (Ciobanu, 2017; Rotaru, 2018).

This study, based on data collected through a questionnaire and an interview guide applied to a sample of 1,039 people released from detention, brings to the fore their perspectives and experiences, highlighting the main challenges and needs. The results of the study show that 30.3% of respondents identified the lack of community support as a major problem, while 17.8% reported difficulties in returning to family and community. Homelessness was mentioned by 8.9% of respondents, and 27.5% highlighted the lack of financial resources as a significant barrier to reintegration. Also, 13.6% of respondents reported a lack of adequate professional qualification, and 22.8% encountered difficulties due to their criminal record.

International studies confirm that community support and access to essential resources, such as housing and employment, are crucial factors for successful reintegration (Travis et al., 2014). Multidimensional interventions that include financial and housing support, vocational training programs, and anti-discrimination measures have been shown to be effective in facilitating reintegration and reducing recidivism (Duwe, 2018; Giguere and Dundes, 2002).

The role of public and private institutions is crucial in this process. Probation services, employment agencies, and non-governmental organizations can play a significant role in providing the necessary support to facilitate reintegration (McNeill and Weaver, 2010; Maruna and Immarigeon, 2004). Closer collaboration between these institutions and local communities is also needed to create an enabling environment for reintegration (Bahr et al., 2010).

The objectives of this study are to identify and analyze the main difficulties and needs of persons released from detention and to formulate recommendations for the development of effective public policies. Through a comprehensive and data-driven approach, this study aims to contribute to improving the process of social reintegration and creating a more inclusive and supportive framework for people returning to the community after detention.

## 2 Literature review

The literature on the social reintegration of people released from prison is vast and diverse, exploring multiple perspectives and determining factors that influence this complex process. This section reviews the relevant literature, highlighting major studies and key findings that contribute to understanding the challenges and solutions for post-prison reintegration.

Discrimination and social marginalization are recognized factors that negatively affect the reintegration of persons released from detention. Petersilia (2003) and Solomon et al. (2008) point out that the stigma associated with criminal records significantly reduces employment opportunities and access to essential services. Studies show that employment discrimination is prevalent and contributes to recidivism, creating a vicious circle of social and economic exclusion (Pager, 2003; Holzer et al., 2006).

Lack of economic resources and stable housing are major barriers to reintegration. Herbert et al. (2015) highlight housing insecurity as a critical factor that negatively affects post-prison stability. Studies show that homeless people are more likely to reoffend, highlighting the need for policies that ensure access to adequate housing (Geller and Curtis, 2011).

Lack of appropriate professional qualifications and criminal record biases are significant barriers to economic reintegration. Bushway et al. (2007) show that lack of adequate training limits employment opportunities, while stigma reduces the chances of getting a job (Holzer et al., 2006). Vocational training programs and employment support interventions are essential for successful reintegration (Duwe, 2018).

Community support and interventions by public and private institutions play a crucial role in facilitating reintegration. International studies suggest that community support programs and multidimensional interventions, which include financial and housing support, have a significant impact on reducing recidivism and improving reintegration outcomes (Travis et al., 2014; McNeill and Weaver, 2010). Bahr et al. (2010) emphasize the importance of collaboration between probation institutions, employment agencies, and non-governmental organizations to create an enabling environment for reintegration.

The literature highlights the complexity of post-prison reintegration, highlighting the need for coordinated and comprehensive interventions to address the multiple barriers faced by persons released from detention. The reviewed studies suggest that effective public policies must include anti-discrimination measures, financial and housing support, vocational training programs, and integrated community interventions to facilitate reintegration and reduce recidivism.

In a study by Bushway et al. (2007), the authors explore the economic and employment barriers faced by ex-convicts in post-industrial America. The study highlights the negative impact of criminal records on employment chances and highlights how stigma reduces access to essential economic opportunities. The authors discuss how bias and discrimination in employment contribute to recidivism, creating a vicious cycle of social and economic exclusion. They propose interventions to improve economic reintegration, including vocational training programs and awareness-raising initiatives for employers to reduce discrimination in employment processes. The study also provides an analysis of existing policies and suggests changes that could facilitate ex-prisoners’ access to the labor market, thus contributing to their economic and social stability.

In a study by Lattimore et al. (2016), the authors analyze the effectiveness of reintegration services for participants in the SVORI assessment, focusing on factors that contribute to successful reintegration and reduce recidivism. The study identifies multidimensional interventions, including financial support, housing and vocational training programs, as essential for facilitating reintegration. The results suggest that community support and access to essential resources can significantly reduce the risk of recidivism and improve the economic and social stability of former prisoners. The authors also highlight the importance of collaboration between probation institutions, employment agencies, and non-governmental organizations to create an enabling environment for reintegration. The study concludes that a coordinated and comprehensive approach can facilitate the effective reintegration of ex-prisoners and help reduce recidivism in the long term.

## 3 Materials and methods

### 3.1 Purpose and objectives

The purpose of this article is to identify and analyze the main difficulties and needs of people released from detention in Romania and to formulate recommendations for the development of effective public policies. Through a comprehensive and data-driven approach, the study aims to contribute to improving the social reintegration process and creating a more inclusive and supportive framework for people returning to the community after detention.

*O1. Identifying the main difficulties faced by people released from detention*: The study aims to identify and analyze the major challenges faced by these people, such as lack of community support, difficulties in returning to family and community, lack of housing and lack of financial resources.

*O2. Analysing the perceptions and needs of people released from detention*: The study aims to understand the perspectives of these people on their reintegration into society, including difficulties in employment and access to essential resources.

*O3. Assessing the impact of socio-economic factors and criminal records on reintegration*: Another objective is to analyse how discrimination, social marginalization and economic hardship influence the reintegration process.

*O4. Formulating recommendations for the development of effective public policies*: The study aims to suggest measures and interventions that address the complex needs of people released from detention, including financial and housing support, vocational training programs, and anti-discrimination measures.

*O5. Contribution to improving the social reintegration process*: By identifying needs and difficulties, the study aims to contribute to the creation of a more inclusive and supportive framework for people released from detention, thus reducing the rate of recidivism.

### 3.2 Assumptions

*Hypothesis 1*: There are differences in the level of community support perceived by inmates depending on the type of penitentiary.

*Hypothesis 2*: There are differences in the difficulties in returning to the family and community depending on the length of the sentence.

*Hypothesis 3*: There are differences in the lack of housing or shelter, depending on the length of the sentence the prisoners received.

*Hypothesis 4*: There are differences in the lack of financial resources after issuance depending on marital status.

*Hypothesis 5*: There are differences in the identification of a job, in general, or of a job according to the qualification acquired and the penitentiary in which the respondents are.

### 3.3 Sample

The studied sample included 1,039 people released from Romanian penitentiaries. Of these, 78% were men and 22% were women. The age of the participants ranged from 18 to 65 years, with an average of 34 years. Most respondents had a low level of education, 45% having only secondary education, 30% high school education and only 25% higher education. From the 1,039 responses to the questionnaire, in order to complete the data obtained through the questionnaire, we also conducted 500 semi—structured interviews. However, we would like to mention that we further present in this paper, only the results of the quantitative study.

### 3.4 Research methods and tools

Data were collected using two main tools: a structured questionnaire and a semi-structured interview guide. The questionnaire was designed to collect quantitative data about post-liberation experiences, while the interview guide was used to obtain more detailed qualitative information about participants’ perceptions and needs.

1. Questionnaire: included questions on difficulties encountered after release, community support, housing situation, access to the labor market and financial resources. The questionnaire was administered in physical and electronic format, depending on the participants’ preferences.
2. Interview guide: semi-structured interviews were conducted to deepen the key aspects identified in the questionnaire. The interviews were conducted by trained researchers and lasted between 30 and 60 min each.

Data collection took place between January and June 2023. Participants were contacted through probation services and non-governmental organizations that provide support to people released from detention. All participants were informed about the purpose and nature of the study, and informed consent was obtained before the questionnaires and interviews began.

The quantitative data collected through the questionnaires were analysed using the Statistical Package for the Social Sciences (SPSS) software. Descriptive analyses were performed to obtain frequencies and percentages, and correlation tests were used to identify relationships between the variables studied.

The qualitative data obtained from the interviews were fully transcribed and analyzed thematically. This method allowed to identify and organize recurring themes and sub-themes relevant to the social reintegration of participants.

## 4 Results

Considering the analysis of the results that is further presented, in our study we did not used dummy variables. However, for the variable “exists” we assigned code 1, and for the variable „does not exist” we assigned code 0. Next, in all the *t*-tests conducted, the first variable analysed had the code 1 and the second variable analysed had the code 2.

### 4.1 Difficulties encountered after release

1. *Lack of community support*: 30.3% of respondents identified the lack of community support as a major problem. This included difficulties in accessing reintegration services and a lack of support from local organizations.

**Figure 1:**
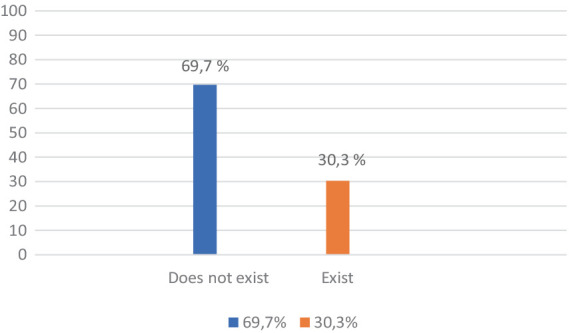
Lack of support from the community, associated with the phenomenon of discrimination.

According to the results in Figure 1, the majority of respondents (69.7%) said they have no difficulties with the support they receive from the community, and 30.3% of them said they have such difficulties. Considering this result, the processing carried out shows an influence of the penitentiary in which the respondents are located on their opinion.

The results presented in Table 1 [*t* (1037) = −1.082, *p* = 0.036 < 0.05], suggests that the persons who are imprisoned in the Poarta Albă penitentiary in Constanta consider to a greater extent than the persons who are imprisoned in the Tulcea penitentiary, that they encounter difficulties in terms of the support they obtain from the community. Thus, it may be that because of the way inmates are treated in the two penitentiaries, or because of the way they keep in touch with friends and family while incarcerated, they may have different opinions regarding the support they will receive from the community after they are released. In other words, this result must be taken into account considering other factors such as distance from family or the way they are treated by prison staff.

**Table 1:** *T*-test for the lack of support from the community, associated with the phenomenon of discrimination and the penitentiary in which the respondents are.

	Group	*N*	Mean	SD	*T*-test for independent samples						
					*t*	df	*p*	Mean difference	Std. error difference	CI4	
	Prison									Lower	Upper
Lack of support from the community	Tulcea	351	0.32	0.46	1.082	1,037	0.036	0.03	0.03	−0.02	0.09
	White Gate Constanta	688	0.29	0.45							

According to this statistical result, the hypothesis that there are differences between the level of support from the community perceived by the detainees depending on the type of penitentiary is confirmed.

2. *Difficulties in returning to family and community*: 17.8% reported difficulties in reintegrating into family and community, citing family tensions and social stigma.

**Figure 2:**
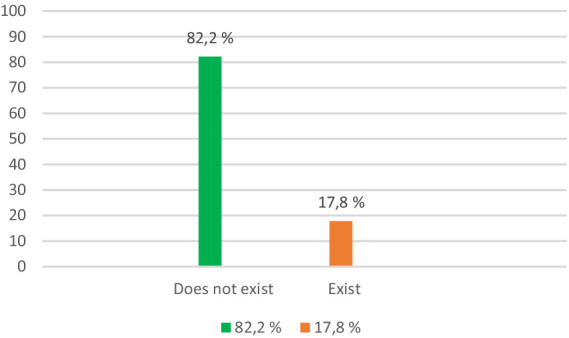
Difficulties in returning to family and community.

According to Figure 2, the majority of respondents (82.2%) said they do not have difficulties in returning to the family and the community, and only 17.8% of them said they have such difficulties. To see how respondents’ opinion differs according to certain variables, we conducted a series of *t*-tests, and the results of the research show differences of opinion depending on the length of punishment that respondents received.

According to the results in Table 2 [*t* (260) = −1.416, *p* = 0.010 < 0.05], the null hypothesis according to which the average of the two variables is equal can be rejected and it can be stated that the persons who received a sentence between 6 and 10 years consider to a greater extent than the persons who received a sentence of more than 20 years, that they have difficulties in reintegrating into the family and the community. In this sense, it is possible that people who spend a longer period of time in prison will get used to managing on their own or will no longer necessarily seek validation from their family and community - this attitude can also be associated with the age at which they will be released (which is older if they receive more than 20 years in prison), while people who receive between 6 and 10 years old, may have a greater desire to reintegrate and lead a normal life in which they are accepted again by their family and community.

**Table 2:** *T*-test for difficulties in returning to the family and community according to the length of the sentence.

	Group	*N*	Mean	SD	*T*-test for independent samples						
					*t*	df	*p*	Mean difference	Std. error difference	CI4	
	The length of the sentence									Lower	Upper
Difficulties in returning to family and community	Between 6 and 10 years	226	0.13	0.34	−1.416	260	0.010	−0.08	0.06	−0.21	0.03
	Over 20 years	36	0.22	0.42							

According to this statistical result, the hypothesis that there are differences in the difficulties in returning to the family and the community depending on the length of the sentence is confirmed.

3. *Homelessness*: 8.9% of respondents cited lack of stable housing as a significant barrier. Most of these people have faced a lack of resources to rent or buy a home.

**Figure 3:**
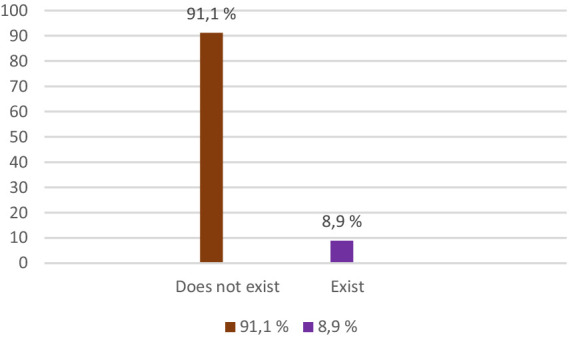
Lack of housing, shelter.

According to the results in Figure 3, most respondents said they do not consider that they will encounter difficulties related to the lack of a home/shelter (91.1%), and only 8.9% of them said that they would encounter such a difficulty.

According to the result of the *t*-test in Table 3 [*t* (264) = −1.672, *p* = 0.002 < 0.05], it is observed that persons who have received a sentence between 6 and 10 years consider to a greater extent than persons who have received a sentence between 16 and 20 years, that they may find it difficult to find shelter or housing after they are released. This result can also be influenced by other factors such as family or friends, which can help the former inmate to obtain housing. Thus, it seems that people who have received higher sentences have greater fears about their reintegration into society from the perspective of obtaining housing.

**Table 3:** *T*-test for homelessness—depending on the length of the sentence.

	Group	*N*	Mean	SD	*T*-test for independent samples						
					*t*	df	*p*	Mean difference	Std. error difference	CI4	
	The length of the sentence									Lower	Upper
Lack of a shelter, of a dwelling	Between 6 and 10 years	226	0.13	0.34	−1.672	264	0.002	−0.08	0.05	−0.18	0.01
	Between 16 and 20 years old	40	0.22	0.42							

According to this statistical result, the hypothesis that there are differences in the lack of a home or shelter is confirmed, depending on the length of the sentence that the detainees received.

4. *Insufficient financial resources*: 27.5% highlighted the lack of financial resources as a major obstacle. This includes difficulties in finding a job due to criminal record biases and a lack of appropriate professional qualifications.

**Figure 4:**
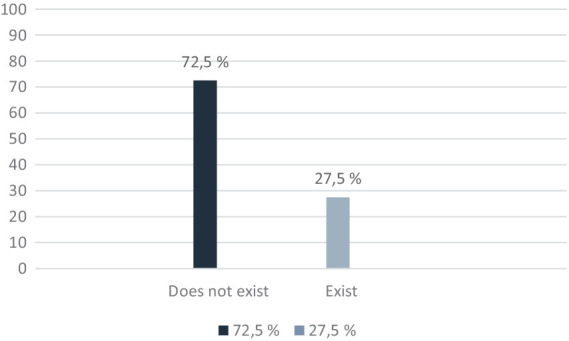
Lack of financial resources after release.

The results presented in Figure 4, shows that most respondents do not believe they will encounter difficulties in terms of financial resources after being released (72.5%), and 27.5% of them said that they may have financial difficulties.

The results of the *t*-test, presented in Table 4 [*t* (568) = 1.033, *p* = 0.038 < 0.05], show that persons who are cohabiting consider to a greater extent than persons who are married, the fact that they will encounter financial difficulties after being released. In this sense, it is possible that married people feel more secure due to the income that their wives have, while people who stay in cohabitation may have the fear that they will not receive financial support from their life partner.

**Table 4:** *T*-test for lack of financial resources after issuance according to marital status.

	Group	*N*	Mean	SD	*T*-test for independent samples						
					*t*	df	*p*	Mean difference	Std. error difference	CI4	
	The length of the sentence									Lower	Upper
Lack of financial resources	married	303	0.25	0.43	1.033	568	0.038	0.03	0.03	−0.03	0.10
	Cohabiting	267	0.22	0.41							

According to this statistical result, the hypothesis that there are differences in the lack of financial resources after issuance depending on the marital status is confirmed.

5. *Professional qualification and access to the labor market*: 13.6% of respondents reported the lack of an adequate professional qualification, and 22.8% encountered difficulties due to the criminal record.

**Figure 5:**
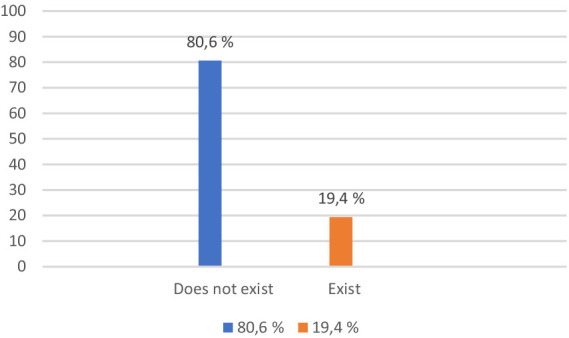
Difficulties in identifying a job, in general, or a job according to the qualification acquired.

According to Figure 5, most respondents do not believe that they will encounter difficulties in finding a job (80.6%), and 19.4% of them mentioned that they may encounter this difficulty after being released.

The results of the *t*-test in Table 5 [*t* (1,037) = −1,200 *p* = 0.015 < 0.05], suggest that the persons who are incarcerated in the Tulcea penitentiary are of the opinion to a greater extent, than the respondents in the Poarta Albă penitentiary Constanța, that they will have difficulties in finding a job after release. It is thus possible that the respondents from the Tulcea penitentiary will be provided with fewer activities to help them maintain or develop certain professional skills, compared to the respondents from the Tulcea penitentiary. However, this result can also be influenced by factors such as the respondents’ residence, the job opportunities they have in the city, the way the community accepts them later.

**Table 5:** *T*-test for difficulties in identifying a job, in general, or a job according to the qualification acquired and the penitentiary in which the respondents are located.

	Group	*N*	Mean	SD	*T*-test for independent samples						
					*t*	df	*p*	Mean difference	Std. error difference	CI4	
	Prison									Lower	Upper
Lack of a job	Tulcea	351	0.17	0.37	−1.200	1,037	0.015	−0.03	0.02	−0.08	0.01
	White Gate Constanta	688	0.20	0.40							

According to this statistical result, the hypothesis according to which there are differences in the identification of a job, in general, or of a job according to the qualification acquired and the penitentiary in which the respondents are located, is confirmed.

## 5 Discussion

The results of this study highlight the complexity of the process of social reintegration of people released from detention in Romania and underline the need for multidimensional interventions to address the barriers encountered. The study identified several key factors influencing reintegration that require special attention in the development of supportive policies and programs.

A significant aspect highlighted by the study is the lack of community support, with 30.3% of respondents identifying this as a major problem. It suggests that interventions that promote social acceptance and reduce stigma are essential. Community support is essential not only for social reintegration, but also for reducing the risk of relapse, as confirmed by the literature.

Difficulties in reintegrating into the family and community, reported by 17.8% of respondents, indicate that interpersonal relationships are often affected by the period of detention. Family tensions and social stigma can exacerbate these difficulties, suggesting that family mediation and post-detention counseling programs could be beneficial.

The lack of stable housing was mentioned by 8.9% of respondents as a significant barrier. This issue is consistent with other studies that emphasize the importance of housing security for social reintegration (Herbert et al., 2015). Without stable housing, people released from detention are at increased risk of recidivism and economic instability.

The lack of financial resources was highlighted by 27.5% of respondents, highlighting the difficulties in finding a job due to prejudices related to the criminal record and the lack of an adequate professional qualification. These results suggest that vocational training and employment support programs are crucial for successful reintegration. It is also necessary to raise awareness among employers in order to reduce discrimination in employment.

Difficulties in finding a job according to the qualification acquired were reported by 22.8% of respondents, emphasizing the importance of adequate professional training programs in prisons. Studies show that access to training and job opportunities can significantly reduce recidivism.

The comparative analysis of the difficulties encountered shows that the type of penitentiary and the length of the sentence have a significant impact on the perception of community support and reintegration difficulties. For example, people who have spent more time in detention or who have been incarcerated in certain penitentiaries report greater difficulties in reintegration, suggesting that experiences in detention can significantly influence the success of reintegration.

The study highlights the crucial role of public and private institutions in facilitating reintegration. Probation services, employment agencies and non-governmental organizations can play a significant role in providing the necessary support. Close collaboration between these institutions and local communities is essential to create an enabling environment for reintegration.

In the process of social reintegration of people released from detention, community involvement is essential to prevent recidivism and facilitate their adaptation to society. Diana Gorea points out that stigma and social judgment are significant barriers to effective reintegration. Thus, probation services play a critical role, providing support for former detainees to lead a responsible life in the community, different from the isolated and restrictive environment of penitentiaries (Gorea, 2022).

Also, Mihai Diţa explores various strategies and methods used by social workers to support ex-prisoners in re-establishing ties with their families. These methods are essential for social reintegration and reducing the risk of relapse, as the family can provide vital support in the rehabilitation process. Diţa highlights the importance of a family-centered approach and the individual needs of the former detainee, emphasizing the role of the social worker in facilitating the reconnection and stabilization of post-detention family relationships (Diţa, 2015).

Based on the results, it is recommended to develop comprehensive public policies that address the complex needs of persons released from detention. These should include financial and housing support, vocational training programs and anti-discrimination measures. Implementing such policies can facilitate effective reintegration and reduce the rate of recidivism.

In conclusion, the social reintegration of people released from detention is a complex process influenced by multiple barriers. The study highlights the need for multidimensional interventions and support from public and private institutions to create a supportive and inclusive framework.

## 6 Limitations

The studied sample includes people released from Romanian penitentiaries, and the results may not be generalizable to other national or international contexts. Cultural, economic and legislative differences can significantly influence the process of social reintegration, which makes it difficult to apply the conclusions of this study in other countries.

Study participants were recruited through probation services and non-governmental organizations, which may introduce a self-selection bias. The people who chose to participate in the study could be those who are already engaged to some extent in the reintegration process and therefore may have different perspectives than those who did not.

The data collected through questionnaires and semi-structured interviews are based on participants’ self-reports, which can introduce memory or reporting errors. Some people may have under- or overestimated the difficulties encountered or the support they received, which can affect the accuracy of the results.

Data collection took place over a period of 6 months, which may not be enough to capture all aspects of the long-term reintegration process. Social reintegration is a long process and some difficulties or successes may arise only after a longer period of time.

This study is cross-sectional, meaning that data was collected at a single point in time. Longitudinal studies, which follow participants over a longer period, could provide more detailed and accurate information about the evolution of social reintegration and the factors influencing its long-term success.

There are many contextual factors, such as the general economic situation, legislative changes and social attitudes, which were not controlled for in this study, but which can significantly influence social reintegration. Also, variations in the rehabilitation and support practices offered by different penitentiaries may affect outcomes.

The questionnaires and interview guides used may have their own limitations in terms of comprehensiveness and accuracy. Some important aspects of attendee experiences may not have been fully captured by these tools.

Despite these limitations, the study provides valuable insights into the challenges and needs of people released from detention in Romania, contributing to understanding the complexity of the social reintegration process and formulating recommendations for effective public policies.

## 7 Conclusion

This study investigated the challenges and needs of people released from detention in Romania, highlighting the multiple barriers they face in the process of social reintegration. Based on data collected from 1,039 respondents through questionnaires and semi-structured interviews, we identified some key conclusions.

Lack of community support was identified as a major problem by 30.3% of respondents. It stresses the need for intervention programs that promote social acceptance and reduce the stigmatization of those released from detention. Public and private institutions, along with non-governmental organizations, play a crucial role in providing this support.

About 17.8% of participants reported difficulties in reintegrating into family and community, citing family tensions and social stigma. These results suggest that family counseling and mediation programs are needed to facilitate reconciliation and social reintegration.

The lack of stable housing was mentioned by 8.9% of respondents, highlighting the need for housing support policies. Ensuring access to adequate housing is essential for post-detention stability and the prevention of recidivism.

Insufficient financial resources were highlighted by 27.5% of respondents, reflecting difficulties in finding a job due to criminal record biases and lack of appropriate professional qualifications. It is crucial to develop vocational training programs and anti-discrimination measures in the labor market to facilitate the employment of these people.

Difficulties in finding suitable employment were reported by 22.8% of participants, which underlines the importance of training programs during detention and initiatives to support post-detention employment. Raising awareness among employers to reduce discrimination in employment is also essential.

The comparative analysis of the difficulties encountered showed that the type of penitentiary and the length of the sentence significantly influence the perception of community support and the difficulties of reintegration. This suggests that experiences in detention and its duration can have a major impact on the success of social reintegration.

In order to facilitate the effective reintegration of persons released from detention and to reduce the rate of recidivism, it is recommended to develop comprehensive public policies that address the complex needs of this population. These should include financial and housing support, vocational training programs, family counseling and anti-discrimination measures.

In conclusion, the social reintegration of people released from detention is a complex process, influenced by multiple barriers. Our study highlights the need for coordinated and multidimensional interventions, supported by public and private institutions, to create a supportive and inclusive framework that facilitates reintegration and contributes to reducing relapse.

## Data Availability

The original contributions presented in the study are included in the article/supplementary material, further inquiries can be directed to the corresponding author.
